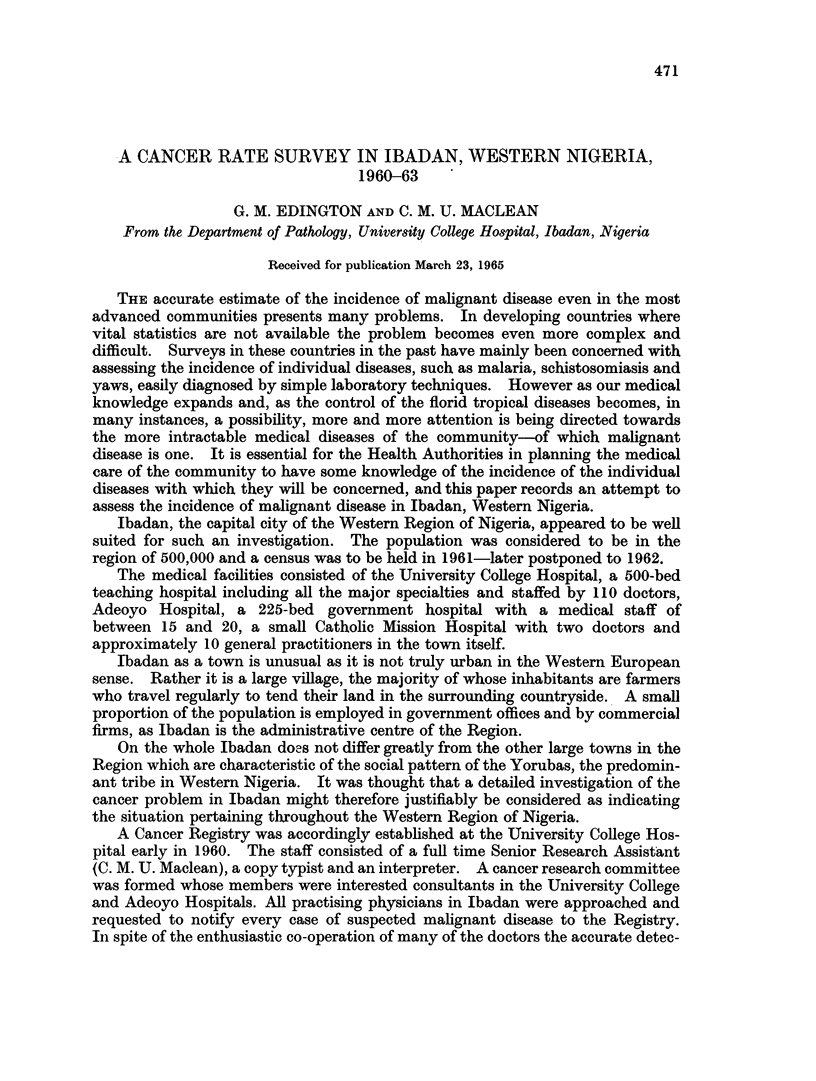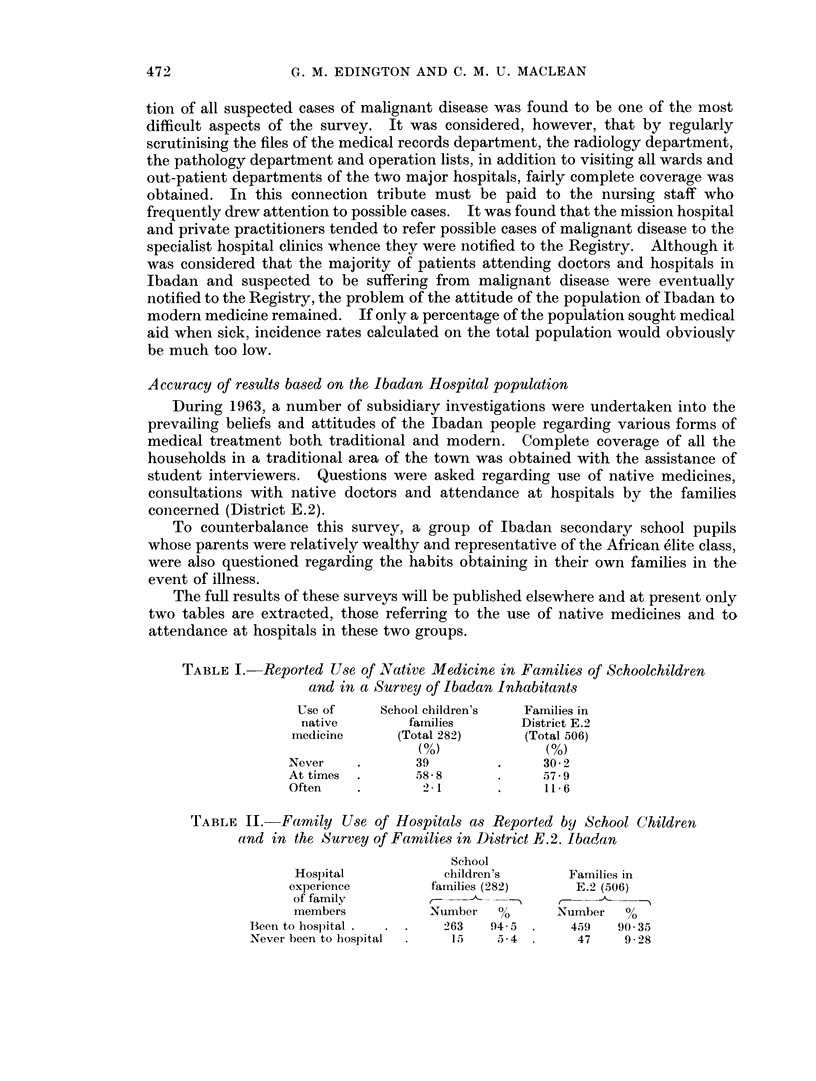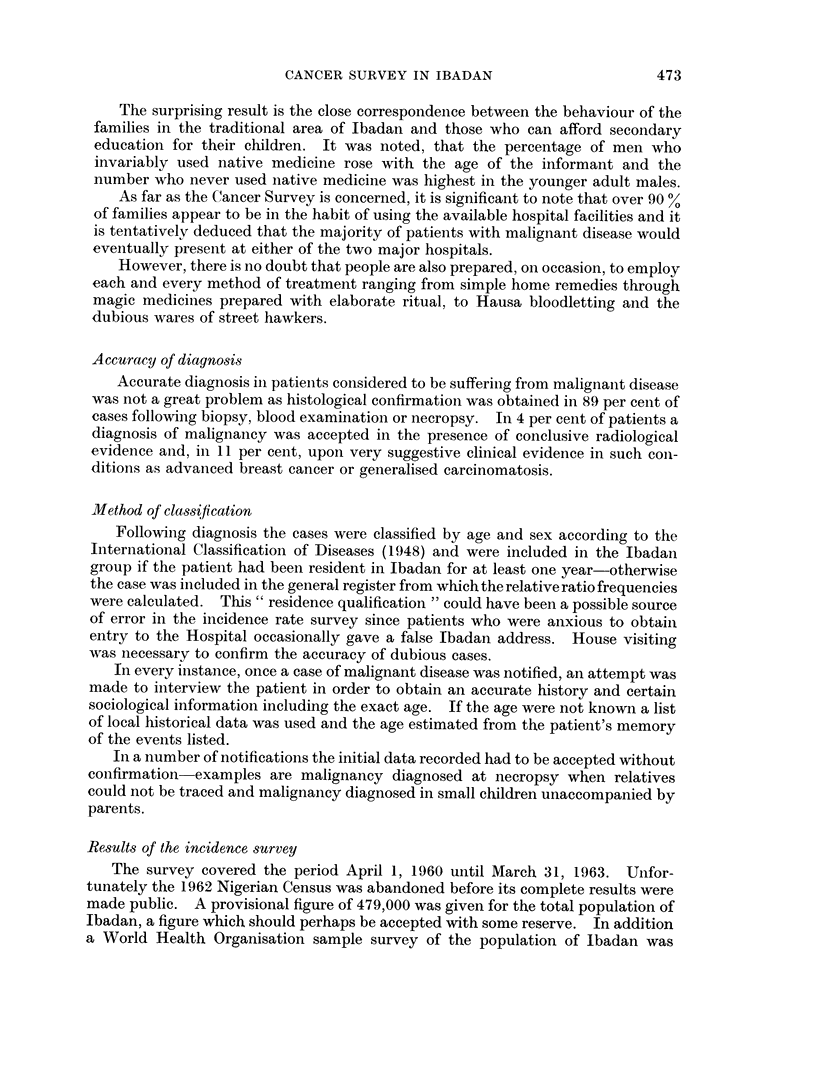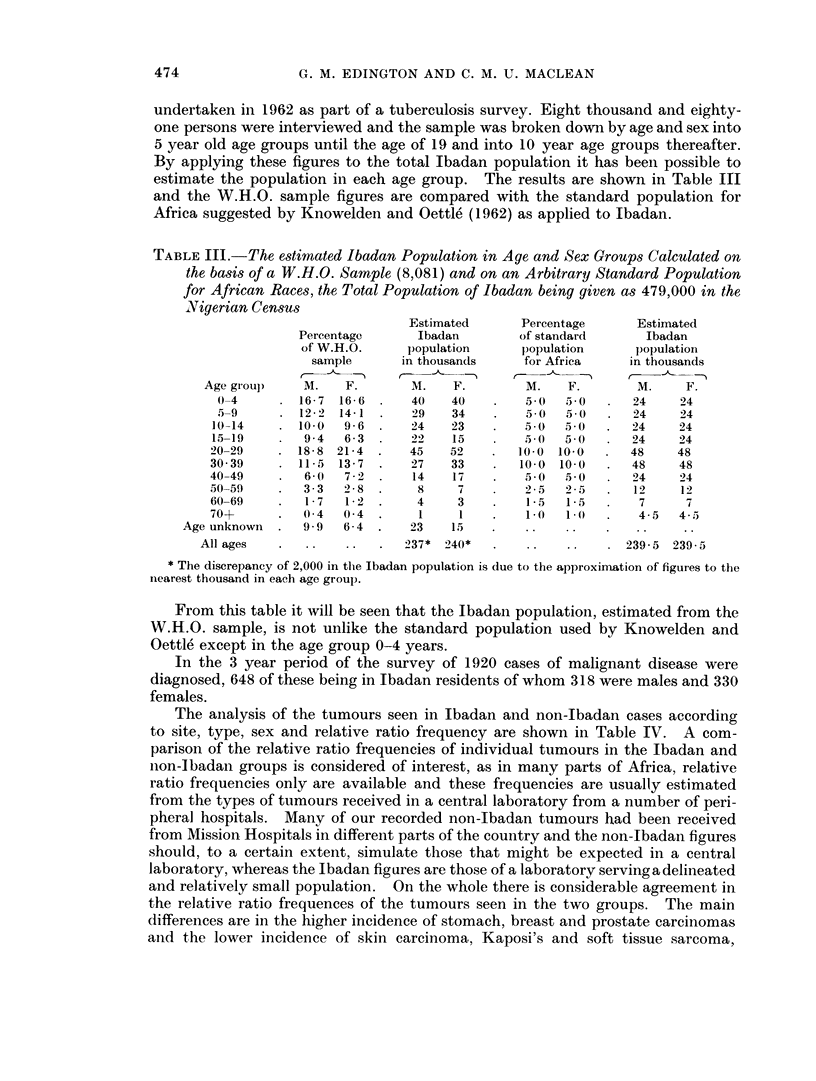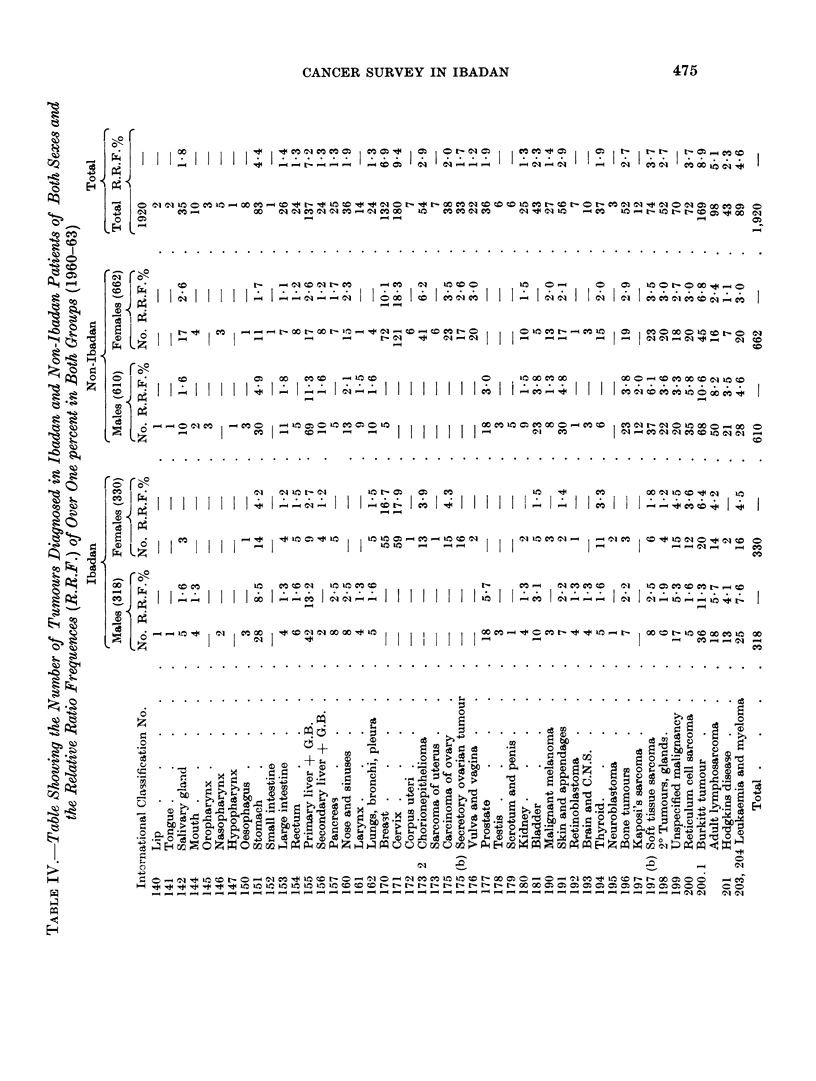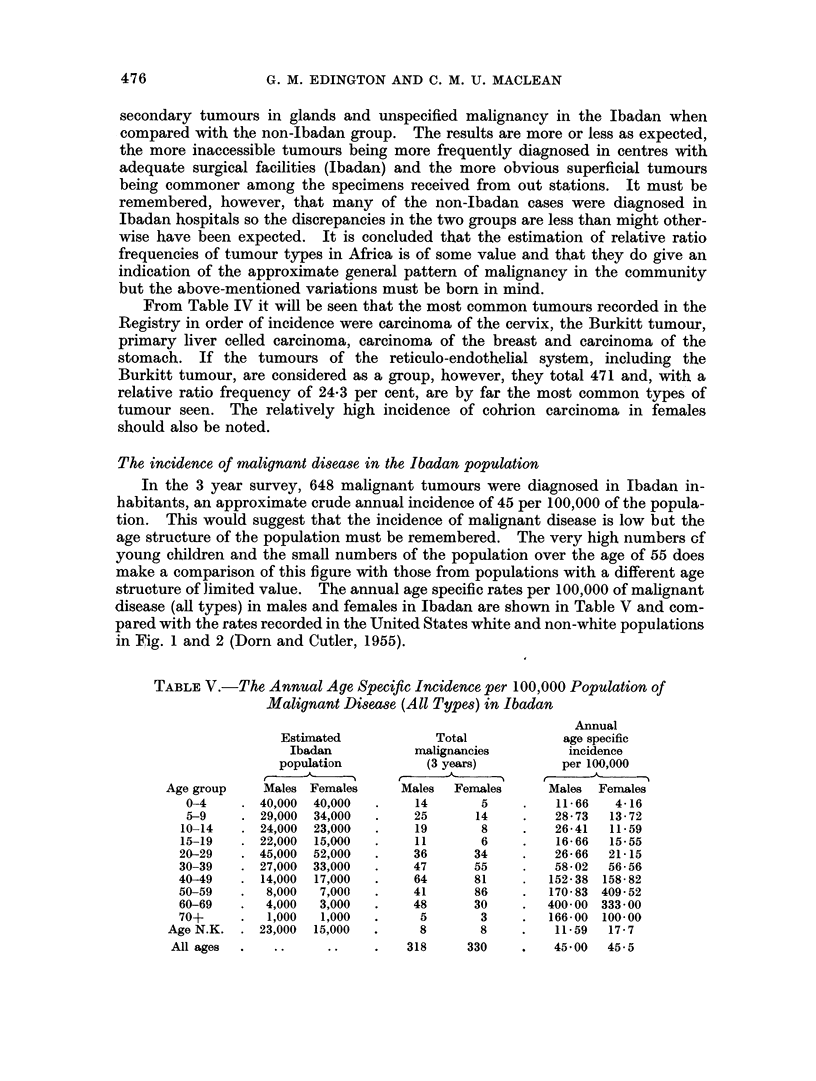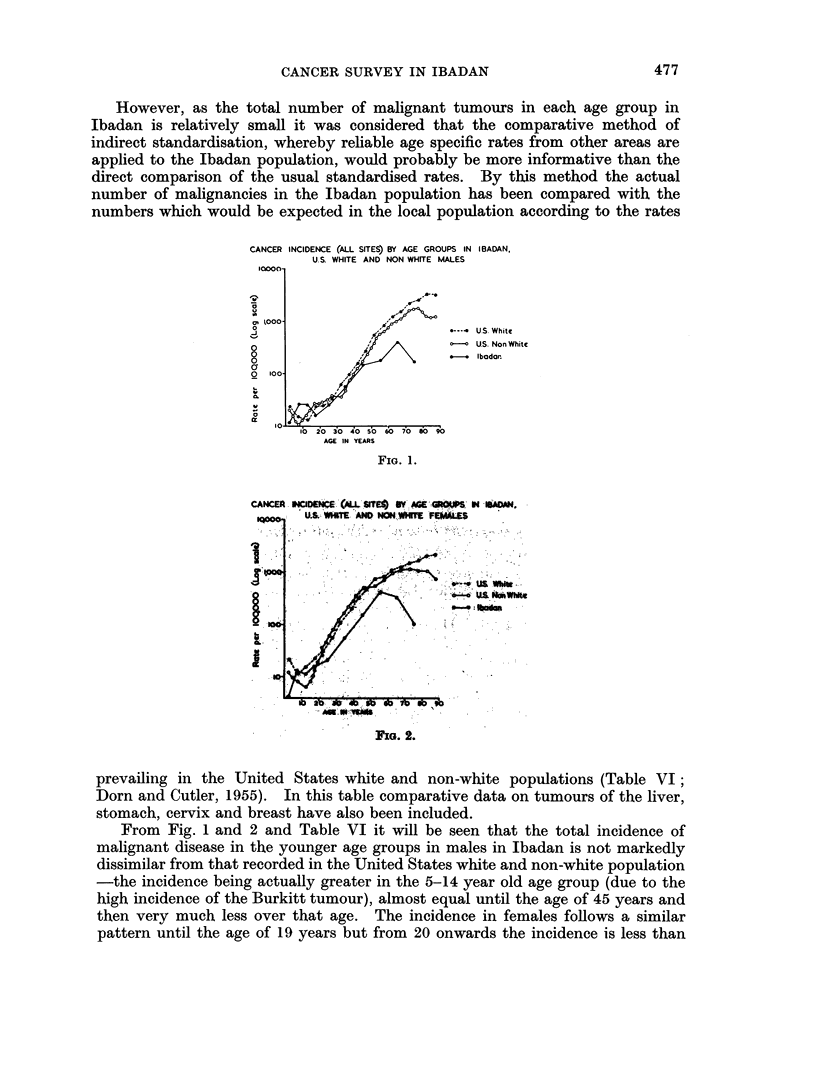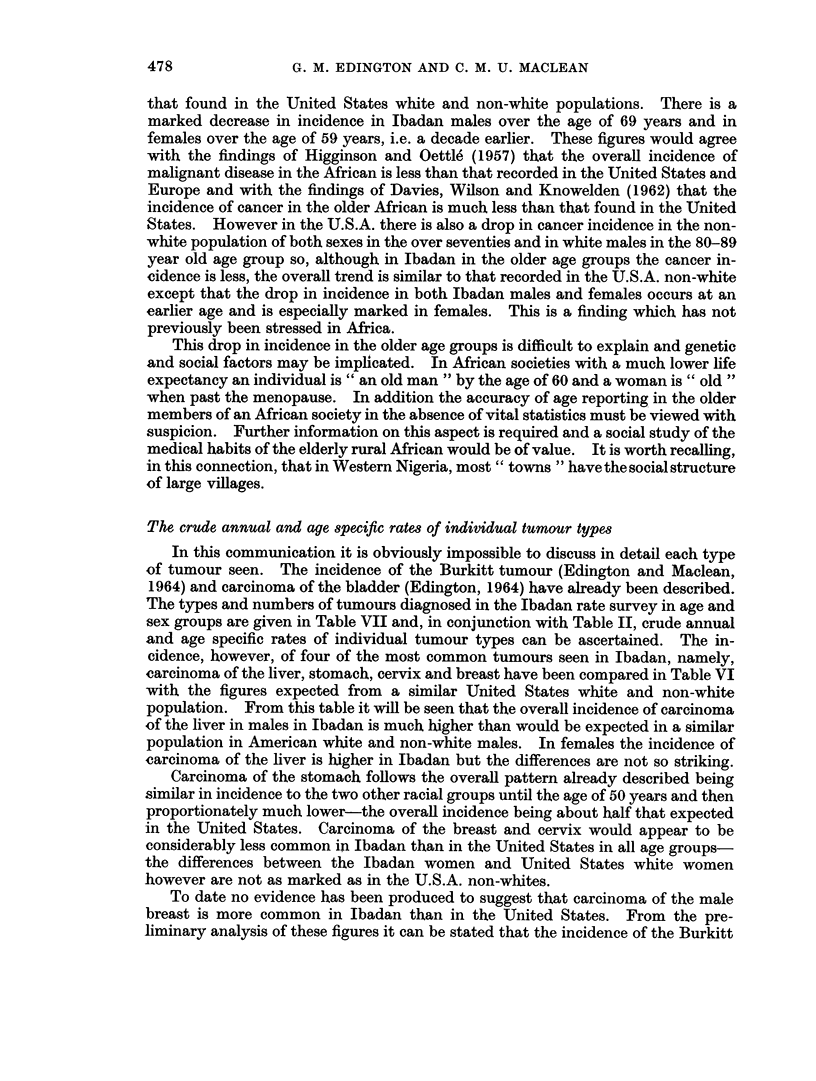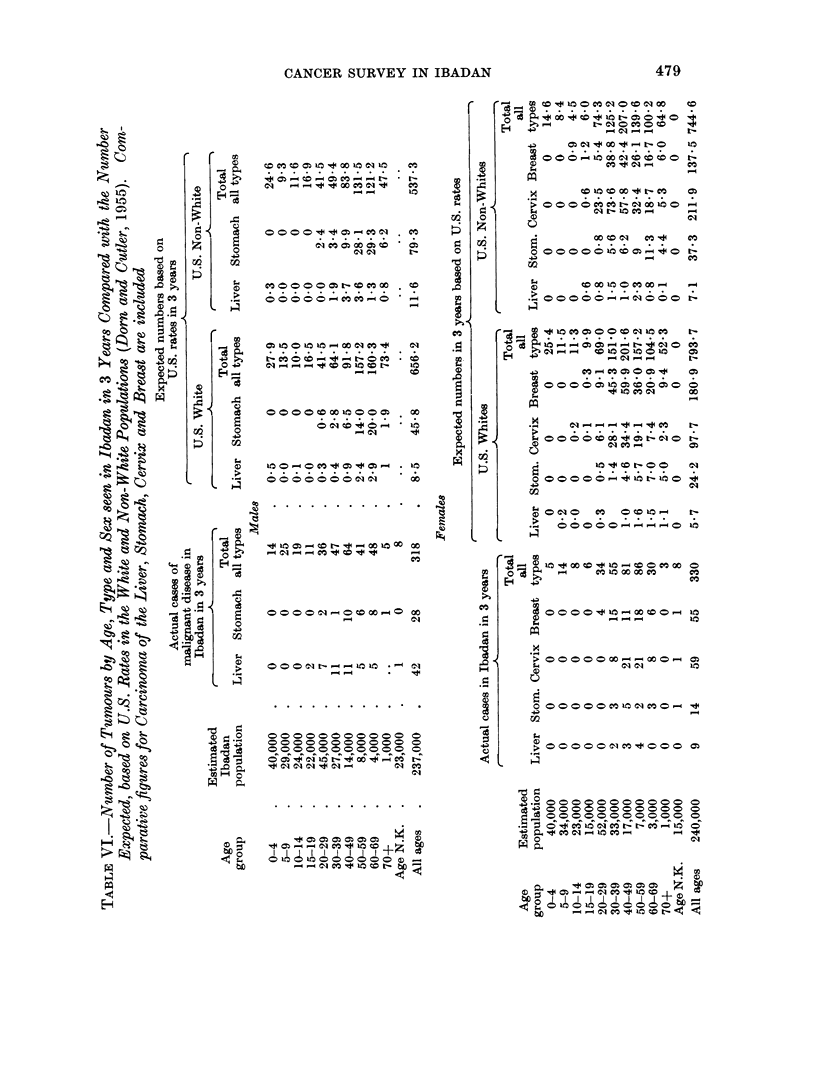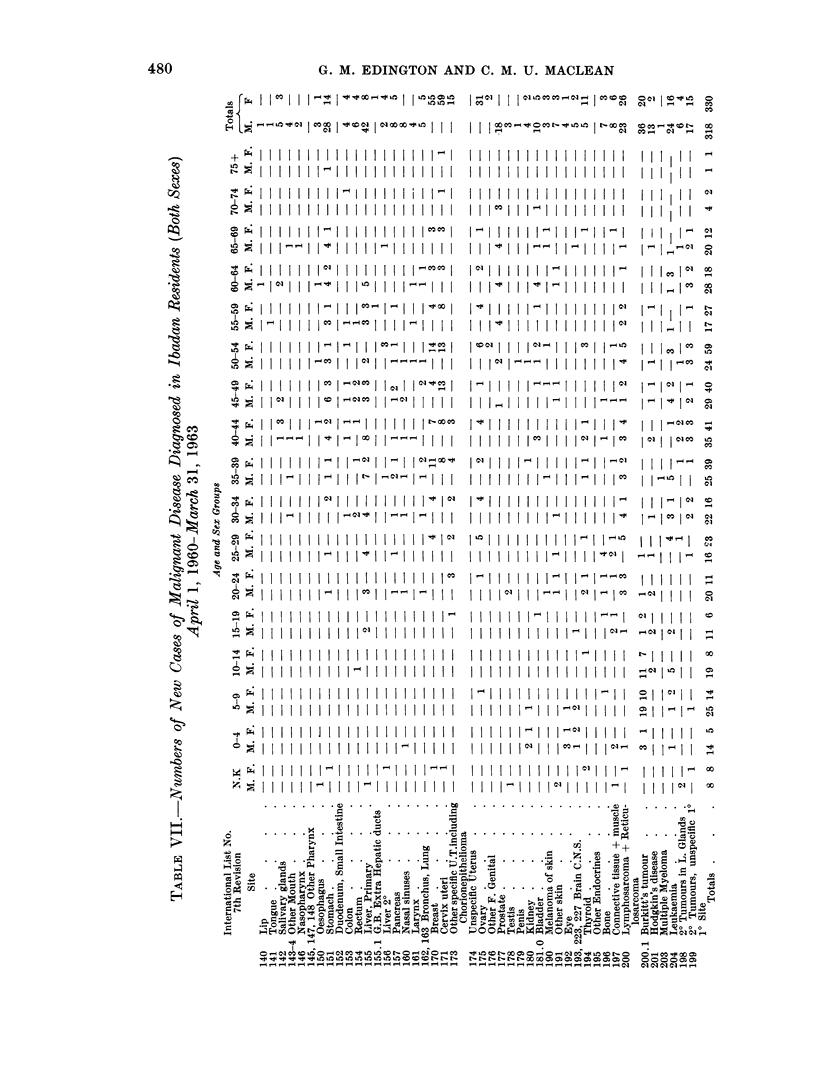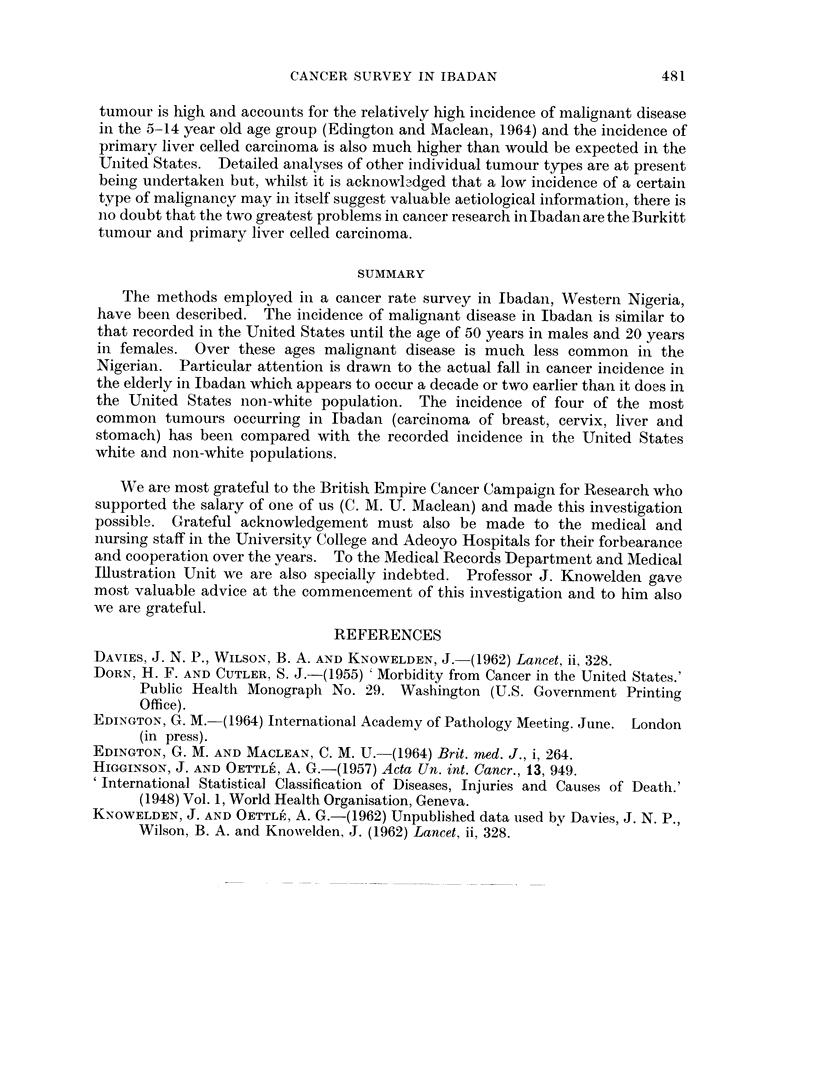# A Cancer Rate Survey in Ibadan, Western Nigeria, 1960-63

**DOI:** 10.1038/bjc.1965.55

**Published:** 1965-09

**Authors:** G. M. Edington, C. M. U. Maclean


### A CANCER RATE SURVEY IN IBADAN, WESTERN NIGERIA, 1960–63

G. M. EDINGTON AND C. M. U. MACLEAN
*From the Department of Pathology, University College Hospital, Ibadan, Nigeria*

Received for publication March 23, 1965

THE accurate estimate of the incidence of malignant disease even in the most advanced communities presents many problems. In developing countries where vital statistics are not available the problem becomes even more complex and difficult. Surveys in these countries in the past have mainly been concerned with assessing the incidence of individual diseases, such as malaria, schistosomiasis and yaws, easily diagnosed by simple laboratory techniques. However as our medical knowledge expands and, as the control of the florid tropical diseases becomes, in many instances, a possibility, more and more attention is being directed towards the more intractable medical diseases of the community—of which malignant disease is one. It is essential for the Health Authorities in planning the medical care of the community to have some knowledge of the incidence of the individual diseases with which they will be concerned, and this paper records an attempt to assess the incidence of malignant disease in Ibadan, Western Nigeria.

Ibadan, the capital city of the Western Region of Nigeria, appeared to be well suited for such an investigation. The population was considered to be in the region of 500,000 and a census was to be held in 1961—later postponed to 1962.

The medical facilities consisted of the University College Hospital, a 500-bed teaching hospital including all the major specialties and staffed by 110 doctors, Adeoyo Hospital, a 225-bed government hospital with a medical staff of between 15 and 20, a small Catholic Mission Hospital with two doctors and approximately 10 general practitioners in the town itself.

Ibadan as a town is unusual as it is not truly urban in the Western European sense. Rather it is a large village, the majority of whose inhabitants are farmers who travel regularly to tend their land in the surrounding countryside. A small proportion of the population is employed in government offices and by commercial firms, as Ibadan is the administrative centre of the Region.

On the whole Ibadan does not differ greatly from the other large towns in the Region which are characteristic of the social pattern of the Yorubas, the predominant tribe in Western Nigeria. It was thought that a detailed investigation of the cancer problem in Ibadan might therefore justifiably be considered as indicating the situation pertaining throughout the Western Region of Nigeria.

A Cancer Registry was accordingly established at the University College Hospital early in 1960. The staff consisted of a full time Senior Research Assistant (C. M. U. Maclean), a copy typist and an interpreter. A cancer research committee was formed whose members were interested consultants in the University College and Adeoyo Hospitals. All practising physicians in Ibadan were approached and requested to notify every case of suspected malignant disease to the Registry. In spite of the enthusiastic co-operation of many of the doctors the accurate detection of all suspected cases of malignant disease was found to be one of the most difficult aspects of the survey. It was considered, however, that by regularly scrutinising the files of the medical records department, the radiology department, the pathology department and operation lists, in addition to visiting all wards and out-patient departments of the two major hospitals, fairly complete coverage was obtained. In this connection tribute must be paid to the nursing staff who frequently drew attention to possible cases. It was found that the mission hospital and private practitioners tended to refer possible cases of malignant disease to the specialist hospital clinics whence they were notified to the Registry. Although it was considered that the majority of patients attending doctors and hospitals in Ibadan and suspected to be suffering from malignant disease were eventually notified to the Registry, the problem of the attitude of the population of Ibadan to modern medicine remained. If only a percentage of the population sought medical aid when sick, incidence rates calculated on the total population would obviously be much too low.

*Accuracy of results based on the Ibadan Hospital population*

During 1963, a number of subsidiary investigations were undertaken into the prevailing beliefs and attitudes of the Ibadan people regarding various forms of medical treatment both traditional and modern. Complete coverage of all the households in a traditional area of the town was obtained with the assistance of student interviewers. Questions were asked regarding use of native medicines, consultations with native doctors and attendance at hospitals by the families concerned (District E.2).

To counterbalance this survey, a group of Ibadan secondary school pupils whose parents were relatively wealthy and representative of the African élite class, were also questioned regarding the habits obtaining in their own families in the event of illness.

The full results of these surveys will be published elsewhere and at present only two tables are extracted, those referring to the use of native medicines and to attendance at hospitals in these two groups.

TABLE I.—*Reported Use of Native Medicine in Families of Schoolchildren and in a Survey of Ibadan Inhabitants*

| Use of native medicine | School children's families (Total 282) (%) | Families in District E.2 (Total 506) (%) |
|---|---|---|
| Never | 39 | 30·2 |
| At times | 58·8 | 57·9 |
| Often | 2·1 | 11·6 |

TABLE II.—*Family Use of Hospitals as Reported by School Children and in the Survey of Families in District E.2. Ibadan*

| Hospital experience of family members | School children's families (282) | | Families in E.2 (506) | |
|---|---|---|---|---|
| | Number | % | Number | % |
| Been to hospital | 263 | 94·5 | 459 | 90·35 |
| Never been to hospital | 15 | 5·4 | 47 | 9·28 |

The surprising result is the close correspondence between the behaviour of the families in the traditional area of Ibadan and those who can afford secondary education for their children. It was noted, that the percentage of men who invariably used native medicine rose with the age of the informant and the number who never used native medicine was highest in the younger adult males.

As far as the Cancer Survey is concerned, it is significant to note that over 90 % of families appear to be in the habit of using the available hospital facilities and it is tentatively deduced that the majority of patients with malignant disease would eventually present at either of the two major hospitals.

However, there is no doubt that people are also prepared, on occasion, to employ each and every method of treatment ranging from simple home remedies through magic medicines prepared with elaborate ritual, to Hausa bloodletting and the dubious wares of street hawkers.

*Accuracy of diagnosis*

Accurate diagnosis in patients considered to be suffering from malignant disease was not a great problem as histological confirmation was obtained in 89 per cent of cases following biopsy, blood examination or necropsy. In 4 per cent of patients a diagnosis of malignancy was accepted in the presence of conclusive radiological evidence and, in 11 per cent, upon very suggestive clinical evidence in such conditions as advanced breast cancer or generalised carcinomatosis.

*Method of classification*

Following diagnosis the cases were classified by age and sex according to the International Classification of Diseases (1948) and were included in the Ibadan group if the patient had been resident in Ibadan for at least one year—otherwise the case was included in the general register from which the relative ratio frequencies were calculated. This " residence qualification " could have been a possible source of error in the incidence rate survey since patients who were anxious to obtain entry to the Hospital occasionally gave a false Ibadan address. House visiting was necessary to confirm the accuracy of dubious cases.

In every instance, once a case of malignant disease was notified, an attempt was made to interview the patient in order to obtain an accurate history and certain sociological information including the exact age. If the age were not known a list of local historical data was used and the age estimated from the patient's memory of the events listed.

In a number of notifications the initial data recorded had to be accepted without confirmation—examples are malignancy diagnosed at necropsy when relatives could not be traced and malignancy diagnosed in small children unaccompanied by parents.

*Results of the incidence survey*

The survey covered the period April 1, 1960 until March 31, 1963. Unfortunately the 1962 Nigerian Census was abandoned before its complete results were made public. A provisional figure of 479,000 was given for the total population of Ibadan, a figure which should perhaps be accepted with some reserve. In addition a World Health Organisation sample survey of the population of Ibadan was undertaken in 1962 as part of a tuberculosis survey. Eight thousand and eighty-one persons were interviewed and the sample was broken down by age and sex into 5 year old age groups until the age of 19 and into 10 year age groups thereafter. By applying these figures to the total Ibadan population it has been possible to estimate the population in each age group. The results are shown in Table III and the W.H.O. sample figures are compared with the standard population for Africa suggested by Knowelden and Oettlé (1962) as applied to Ibadan.

TABLE III.—*The estimated Ibadan Population in Age and Sex Groups Calculated on the basis of a W.H.O. Sample* (8,081) *and on an Arbitrary Standard Population for African Races, the Total Population of Ibadan being given as* 479,000 *in the Nigerian Census*

| | Percentage of W.H.O. sample | | Estimated Ibadan population in thousands | | Percentage of standard population for Africa | | Estimated Ibadan population in thousands | |
|---|---|---|---|---|---|---|---|---|
| Age group | M. | F. | M. | F. | M. | F. | M. | F. |
| 0–4 | 16·7 | 16·6 | 40 | 40 | 5·0 | 5·0 | 24 | 24 |
| 5–9 | 12·2 | 14·1 | 29 | 34 | 5·0 | 5·0 | 24 | 24 |
| 10–14 | 10·0 | 9·6 | 24 | 23 | 5·0 | 5·0 | 24 | 24 |
| 15–19 | 9·4 | 6·3 | 22 | 15 | 5·0 | 5·0 | 24 | 24 |
| 20–29 | 18·8 | 21·4 | 45 | 52 | 10·0 | 10·0 | 48 | 48 |
| 30·39 | 11·5 | 13·7 | 27 | 33 | 10·0 | 10·0 | 48 | 48 |
| 40–49 | 6·0 | 7·2 | 14 | 17 | 5·0 | 5·0 | 24 | 24 |
| 50–59 | 3·3 | 2·8 | 8 | 7 | 2·5 | 2·5 | 12 | 12 |
| 60–69 | 1·7 | 1·2 | 4 | 3 | 1·5 | 1·5 | 7 | 7 |
| 70+ | 0·4 | 0·4 | 1 | 1 | 1·0 | 1·0 | 4·5 | 4·5 |
| Age unknown | 9·9 | 6·4 | 23 | 15 | .. | .. | .. | .. |
| All ages | .. | .. | 237* | 240* | .. | .. | 239·5 | 239·5 |

* The discrepancy of 2,000 in the Ibadan population is due to the approximation of figures to the nearest thousand in each age group.

From this table it will be seen that the Ibadan population, estimated from the W.H.O. sample, is not unlike the standard population used by Knowelden and Oettlé except in the age group 0–4 years.

In the 3 year period of the survey of 1920 cases of malignant disease were diagnosed, 648 of these being in Ibadan residents of whom 318 were males and 330 females.

The analysis of the tumours seen in Ibadan and non-Ibadan cases according to site, type, sex and relative ratio frequency are shown in Table IV. A comparison of the relative ratio frequencies of individual tumours in the Ibadan and non-Ibadan groups is considered of interest, as in many parts of Africa, relative ratio frequencies only are available and these frequencies are usually estimated from the types of tumours received in a central laboratory from a number of peripheral hospitals. Many of our recorded non-Ibadan tumours had been received from Mission Hospitals in different parts of the country and the non-Ibadan figures should, to a certain extent, simulate those that might be expected in a central laboratory, whereas the Ibadan figures are those of a laboratory serving a delineated and relatively small population. On the whole there is considerable agreement in the relative ratio frequences of the tumours seen in the two groups. The main differences are in the higher incidence of stomach, breast and prostate carcinomas and the lower incidence of skin carcinoma, Kaposi's and soft tissue sarcoma,

TABLE IV.—*Table Showing the Number of Tumours Diagnosed in Ibadan and Non-Ibadan Patients of Both Sexes and the Relative Ratio Frequences (R.R.F.) of Over One percent in Both Groups* (1960–63)

| | | Ibadan | | | | Non-Ibadan | | | | Total | |
|---|---|---|---|---|---|---|---|---|---|---|---|
| | | Males (318) | | Females (330) | | Males (610) | | Females (662) | | Total | R.R.F.% |
| International Classification No. | | No. | R.R.F.% | No. | R.R.F.% | No. | R.R.F.% | No. | R.R.F.% | 1920 | — |
| 140 | Lip | 1 | — | — | — | 1 | — | — | — | 2 | — |
| 141 | Tongue | 1 | — | — | — | 1 | — | — | — | 2 | — |
| 142 | Salivary gland | 5 | 1·6 | 3 | — | 10 | 1·6 | 17 | 2·6 | 35 | 1·8 |
| 144 | Mouth | 4 | 1·3 | — | — | 2 | — | 4 | — | 10 | — |
| 145 | Oropharynx | — | — | — | — | 3 | — | — | — | 3 | — |
| 146 | Nasopharynx | 2 | — | — | — | — | — | 3 | — | 5 | — |
| 147 | Hypopharynx | — | — | — | — | 1 | — | — | — | 1 | — |
| 150 | Oesophagus | 3 | — | 1 | — | 3 | — | 1 | — | 8 | — |
| 151 | Stomach | 28 | 8·5 | 14 | 4·2 | 30 | 4·9 | 11 | 1·7 | 83 | 4·4 |
| 152 | Small intestine | — | — | — | — | — | — | 1 | — | 1 | — |
| 153 | Large intestine | 4 | 1·3 | 4 | 1·2 | 11 | 1·8 | 7 | 1·1 | 26 | 1·4 |
| 154 | Rectum | 6 | 1·6 | 5 | 1·5 | 5 | — | 8 | 1·2 | 24 | 1·3 |
| 155 | Primary liver + G.B. | 42 | 13·2 | 9 | 2·7 | 69 | 11·3 | 17 | 2·6 | 137 | 7·2 |
| 156 | Secondary liver + G.B. | 2 | — | 4 | 1·2 | 10 | 1·6 | 8 | 1·2 | 24 | 1·3 |
| 157 | Pancreas | 8 | 2·5 | 5 | — | 5 | — | 7 | 1·7 | 25 | 1·3 |
| 160 | Nose and sinuses | 8 | 2·5 | — | — | 13 | 2·1 | 15 | 2·3 | 36 | 1·9 |
| 161 | Larynx | 4 | 1·3 | — | — | 9 | 1·5 | 1 | — | 14 | — |
| 162 | Lungs, bronchi, pleura | 5 | 1·6 | 5 | 1·5 | 10 | 1·6 | 4 | — | 24 | 1·3 |
| 170 | Breast | — | — | 55 | 16·7 | 5 | — | 72 | 10·1 | 132 | 6·9 |
| 171 | Cervix | — | — | 59 | 17·9 | — | — | 121 | 18·3 | 180 | 9·4 |
| 172 | Corpus uteri | — | — | 1 | — | — | — | 6 | — | 7 | — |
| 173 2 | Chorionepithelioma | — | — | 13 | 3·9 | — | — | 41 | 6·2 | 54 | 2·9 |
| 173 | Sarcoma of uterus | — | — | 1 | — | — | — | 6 | — | 7 | — |
| 175 | Carcinoma of ovary | — | — | 15 | 4.3 | — | — | 23 | 3·5 | 38 | 2·0 |
| 175 (b) | Secretory ovarian tumour | — | — | 16 | — | — | — | 17 | 2·6 | 33 | 1·7 |
| 176 | Vulva and vagina | — | — | 2 | — | — | — | 20 | 3·0 | 22 | 1·2 |
| 177 | Prostate | 18 | 5·7 | — | — | 18 | 3·0 | — | — | 36 | 1·9 |
| 178 | Testis | 3 | — | — | — | 3 | — | — | — | 6 | — |
| 179 | Scrotum and penis | 1 | — | — | — | 5 | — | — | — | 6 | — |
| 180 | Kidney | 4 | 1·3 | 2 | — | 9 | 1·5 | 10 | 1·5 | 25 | 1·3 |
| 181 | Bladder | 10 | 3·1 | 5 | 1·5 | 23 | 3·8 | 5 | — | 43 | 2·3 |
| 190 | Malignant melanoma | 3 | — | 3 | — | 8 | 1·3 | 13 | 2·0 | 27 | 1·4 |
| 191 | Skin and appendages | 7 | 2·2 | 2 | 1·4 | 30 | 4·8 | 17 | 2·1 | 56 | 2·9 |
| 192 | Retinoblastoma | 4 | 1·3 | 1 | — | 1 | — | 1 | — | 7 | — |
| 193 | Brain and C.N.S. | 4 | 1·3 | — | — | 3 | — | 3 | — | 10 | — |
| 194 | Thyroid | 5 | 1·6 | 11 | 3·3 | 6 | — | 15 | 2·0 | 37 | 1·9 |
| 195 | Neuroblastoma | 1 | — | 2 | — | — | — | — | — | 3 | — |
| 196 | Bone tumours | 7 | 2·2 | 3 | — | 23 | 3·8 | 19 | 2·9 | 52 | 2·7 |
| 197 | Kaposi's sarcoma | — | — | — | — | 12 | 2·0 | — | — | 12 | — |
| 197 (b) | Soft tissue sarcoma | 8 | 2·5 | 6 | 1·8 | 37 | 6·1 | 23 | 3·5 | 74 | 3·7 |
| 198 | 2° Tumours, glands. | 6 | 1·9 | 4 | 1·2 | 22 | 3·6 | 20 | 3·0 | 52 | 2·7 |
| 199 | Unspecified malignancy | 17 | 5·3 | 15 | 4·5 | 20 | 3·3 | 18 | 2·7 | 70 | — |
| 200 | Reticulum cell sarcoma | 5 | 1·6 | 12 | 3·6 | 35 | 5·8 | 20 | 3·0 | 72 | 3·7 |
| 200.1 | Burkitt tumour | 36 | 11·3 | 20 | 6·4 | 68 | 10·6 | 45 | 6·8 | 169 | 8·9 |
| | Adult lymphosarcoma | 18 | 5·7 | 14 | 4·2 | 50 | 8·2 | 16 | 2·4 | 98 | 5·1 |
| 201 | Hodgkins disease | 13 | 4·1 | 2 | — | 21 | 3·5 | 7 | 1·1 | 43 | 2·3 |
| 203, 204 | Leukaemia and myeloma | 25 | 7·6 | 16 | 4·5 | 28 | 4·6 | 20 | 3·0 | 89 | 4·6 |
| | Total | 318 | — | 330 | — | 610 | — | 662 | — | 1,920 | — |

secondary tumours in glands and unspecified malignancy in the Ibadan when compared with the non-Ibadan group. The results are more or less as expected, the more inaccessible tumours being more frequently diagnosed in centres with adequate surgical facilities (Ibadan) and the more obvious superficial tumours being commoner among the specimens received from out stations. It must be remembered, however, that many of the non-Ibadan cases were diagnosed in Ibadan hospitals so the discrepancies in the two groups are less than might otherwise have been expected. It is concluded that the estimation of relative ratio frequencies of tumour types in Africa is of some value and that they do give an indication of the approximate general pattern of malignancy in the community but the above-mentioned variations must be born in mind.

From Table IV it will be seen that the most common tumours recorded in the Registry in order of incidence were carcinoma of the cervix, the Burkitt tumour, primary liver celled carcinoma, carcinoma of the breast and carcinoma of the stomach. If the tumours of the reticulo-endothelial system, including the Burkitt tumour, are considered as a group, however, they total 471 and, with a relative ratio frequency of 24·3 per cent, are by far the most common types of tumour seen. The relatively high incidence of cohrion carcinoma in females should also be noted.

*The incidence of malignant disease in the Ibadan population*

In the 3 year survey, 648 malignant tumours were diagnosed in Ibadan inhabitants, an approximate crude annual incidence of 45 per 100,000 of the population. This would suggest that the incidence of malignant disease is low but the age structure of the population must be remembered. The very high numbers of young children and the small numbers of the population over the age of 55 does make a comparison of this figure with those from populations with a different age structure of limited value. The annual age specific rates per 100,000 of malignant disease (all types) in males and females in Ibadan are shown in Table V and compared with the rates recorded in the United States white and non-white populations in Fig. 1 and 2 (Dorn and Cutler, 1955).

TABLE V.—*The Annual Age Specific Incidence per* 100,000 *Population of Malignant Disease* (*All Types*) *in Ibadan*

| | Estimated Ibadan population | | Total malignancies (3 years) | | Annual age specific incidence per 100,000 | |
|---|---|---|---|---|---|---|
| Age group | Males | Females | Males | Females | Males | Females |
| 0–4 | 40,000 | 40,000 | 14 | 5 | 11·66 | 4·16 |
| 5–9 | 29,000 | 34,000 | 25 | 14 | 28·73 | 13·72 |
| 10–14 | 24,000 | 23,000 | 19 | 8 | 26·41 | 11·59 |
| 15–19 | 22,000 | 15,000 | 11 | 6 | 16·66 | 15·55 |
| 20–29 | 45,000 | 52,000 | 36 | 34 | 26·66 | 21·15 |
| 30–39 | 27,000 | 33,000 | 47 | 55 | 58·02 | 56·56 |
| 40–49 | 14,000 | 17,000 | 64 | 81 | 152·38 | 158·82 |
| 50–59 | 8,000 | 7,000 | 41 | 86 | 170·83 | 409·52 |
| 60–69 | 4,000 | 3,000 | 48 | 30 | 400·00 | 333·00 |
| 70+ | 1,000 | 1,000 | 5 | 3 | 166·00 | 100·00 |
| Age N.K. | 23,000 | 15,000 | 8 | 8 | 11·59 | 17·7 |
| All ages | .. | .. | 318 | 330 | 45·00 | 45·5 |

However, as the total number of malignant tumours in each age group in Ibadan is relatively small it was considered that the comparative method of indirect standardisation, whereby reliable age specific rates from other areas are applied to the Ibadan population, would probably be more informative than the direct comparison of the usual standardised rates. By this method the actual number of malignancies in the Ibadan population has been compared with the numbers which would be expected in the local population according to the rates

FIG. 1.

FIG. 2.

prevailing in the United States white and non-white populations (Table VI; Dorn and Cutler, 1955). In this table comparative data on tumours of the liver, stomach, cervix and breast have also been included.

From Fig. 1 and 2 and Table VI it will be seen that the total incidence of malignant disease in the younger age groups in males in Ibadan is not markedly dissimilar from that recorded in the United States white and non-white population —the incidence being actually greater in the 5–14 year old age group (due to the high incidence of the Burkitt tumour), almost equal until the age of 45 years and then very much less over that age. The incidence in females follows a similar pattern until the age of 19 years but from 20 onwards the incidence is less than that found in the United States white and non-white populations. There is a marked decrease in incidence in Ibadan males over the age of 69 years and in females over the age of 59 years, i.e. a decade earlier. These figures would agree with the findings of Higginson and Oettlé (1957) that the overall incidence of malignant disease in the African is less than that recorded in the United States and Europe and with the findings of Davies, Wilson and Knowelden (1962) that the incidence of cancer in the older African is much less than that found in the United States. However in the U.S.A. there is also a drop in cancer incidence in the non-white population of both sexes in the over seventies and in white males in the 80–89 year old age group so, although in Ibadan in the older age groups the cancer incidence is less, the overall trend is similar to that recorded in the U.S.A. non-white except that the drop in incidence in both Ibadan males and females occurs at an earlier age and is especially marked in females. This is a finding which has not previously been stressed in Africa.

This drop in incidence in the older age groups is difficult to explain and genetic and social factors may be implicated. In African societies with a much lower life expectancy an individual is " an old man " by the age of 60 and a woman is " old " when past the menopause. In addition the accuracy of age reporting in the older members of an African society in the absence of vital statistics must be viewed with suspicion. Further information on this aspect is required and a social study of the medical habits of the elderly rural African would be of value. It is worth recalling, in this connection, that in Western Nigeria, most " towns " have the social structure of large villages.

*The crude annual and age specific rates of individual tumour types*

In this communication it is obviously impossible to discuss in detail each type of tumour seen. The incidence of the Burkitt tumour (Edington and Maclean, 1964) and carcinoma of the bladder (Edington, 1964) have already been described. The types and numbers of tumours diagnosed in the Ibadan rate survey in age and sex groups are given in Table VII and, in conjunction with Table II, crude annual and age specific rates of individual tumour types can be ascertained. The incidence, however, of four of the most common tumours seen in Ibadan, namely, carcinoma of the liver, stomach, cervix and breast have been compared in Table VI with the figures expected from a similar United States white and non-white population. From this table it will be seen that the overall incidence of carcinoma of the liver in males in Ibadan is much higher than would be expected in a similar population in American white and non-white males. In females the incidence of carcinoma of the liver is higher in Ibadan but the differences are not so striking.

Carcinoma of the stomach follows the overall pattern already described being similar in incidence to the two other racial groups until the age of 50 years and then proportionately much lower—the overall incidence being about half that expected in the United States. Carcinoma of the breast and cervix would appear to be considerably less common in Ibadan than in the United States in all age groups—the differences between the Ibadan women and United States white women however are not as marked as in the U.S.A. non-whites.

To date no evidence has been produced to suggest that carcinoma of the male breast is more common in Ibadan than in the United States. From the preliminary analysis of these figures it can be stated that the incidence of the Burkitt

TABLE VI.—*Number of Tumours by Age, Type and Sex seen in Ibadan in 3 Years Compared with the Number Expected, based on U.S. Rates in the White and Non-White Populations (Dorn and Cutler,* 1955). *Comparative figures for Carcinoma of the Liver, Stomach, Cervix and Breast are included*

*Males*

| Age group | Estimated Ibadan population | Actual cases of malignant disease in Ibadan in 3 years: Liver | Stomach | Total all types | Expected numbers based on U.S. rates in 3 years — U.S. White: Liver | Stomach | Total all types | U.S. Non-White: Liver | Stomach | Total all types |
|---|---|---|---|---|---|---|---|---|---|---|
| 0–4 | 40,000 | 0 | 0 | 14 | 0·5 | 0 | 27·9 | 0·3 | 0 | 24·6 |
| 5–9 | 29,000 | 0 | 0 | 25 | 0·0 | 0 | 13·5 | 0·0 | 0 | 9·3 |
| 10–14 | 24,000 | 0 | 0 | 19 | 0·1 | 0 | 10·0 | 0·0 | 0 | 11·6 |
| 15–19 | 22,000 | 2 | 0 | 11 | 0·0 | 0 | 16·5 | 0·0 | 0 | 16·9 |
| 20–29 | 45,000 | 7 | 2 | 36 | 0·3 | 0·6 | 41·5 | 0·0 | 2·4 | 41·5 |
| 30–39 | 27,000 | 11 | 1 | 47 | 0·4 | 2·8 | 64·1 | 1·9 | 3·4 | 49·4 |
| 40–49 | 14,000 | 11 | 10 | 64 | 0·9 | 6·5 | 91·8 | 3·7 | 9·9 | 83·8 |
| 50–59 | 8,000 | 5 | 6 | 41 | 2·4 | 14·0 | 157·2 | 3·6 | 28·1 | 131·5 |
| 60–69 | 4,000 | 5 | 8 | 48 | 2·9 | 20·0 | 160·3 | 1·3 | 29·3 | 121·2 |
| 70+ | 1,000 | .. | 1 | 5 | 1 | 1·9 | 73·4 | 0·8 | 6·2 | 47·5 |
| Age N.K. | 23,000 | 1 | 0 | 8 | .. | .. | .. | .. | .. | .. |
| All ages | 237,000 | 42 | 28 | 318 | 8·5 | 45·8 | 656·2 | 11·6 | 79·3 | 537·3 |

*Females*

| Age group | Estimated population | Actual cases in Ibadan in 3 years: Liver | Stom. | Cervix | Breast | Total all types | Expected numbers in 3 years based on U.S. rates — U.S. Whites: Liver | Stom. | Cervix | Breast | Total all types | U.S. Non-Whites: Liver | Stom. | Cervix | Breast | Total all types |
|---|---|---|---|---|---|---|---|---|---|---|---|---|---|---|---|---|
| 0–4 | 40,000 | 0 | 0 | 0 | 0 | 5 | 0 | 0 | 0 | 0 | 25·4 | 0 | 0 | 0 | 0 | 14·6 |
| 5–9 | 34,000 | 0 | 0 | 0 | 0 | 14 | 0·2 | 0 | 0 | 0 | 11·5 | 0 | 0 | 0 | 0 | 8·4 |
| 10–14 | 23,000 | 0 | 0 | 0 | 0 | 8 | 0·0 | 0 | 0·2 | 0 | 11·3 | 0 | 0 | 0 | 0·9 | 4·5 |
| 15–19 | 15,000 | 0 | 0 | 0 | 0 | 6 | 0 | 0 | 0·1 | 0·3 | 9·9 | 0·6 | 0 | 0·6 | 1·2 | 6·0 |
| 20–29 | 52,000 | 0 | 0 | 0 | 4 | 34 | 0·3 | 0·5 | 6·1 | 9·1 | 69·0 | 0·8 | 0·8 | 23·5 | 5·4 | 74·3 |
| 30–39 | 33,000 | 2 | 3 | 8 | 15 | 55 | 0 | 1·4 | 28·1 | 45·3 | 151·0 | 1·5 | 5·6 | 73·6 | 38·8 | 125·2 |
| 40–49 | 17,000 | 3 | 5 | 21 | 11 | 81 | 1·0 | 4·6 | 34·4 | 59·9 | 201·6 | 1·0 | 6·2 | 57·8 | 42·4 | 207·0 |
| 50–59 | 7,000 | 4 | 2 | 21 | 18 | 86 | 1·6 | 5·7 | 19·1 | 36·0 | 157·2 | 2·3 | 9 | 32·4 | 26·1 | 139·6 |
| 60–69 | 3,000 | 0 | 3 | 8 | 6 | 30 | 1·5 | 7·0 | 7·4 | 20·9 | 104·5 | 0·8 | 11·3 | 18·7 | 16·7 | 100·2 |
| 70+ | 1,000 | 0 | 0 | 0 | 0 | 3 | 1·1 | 5·0 | 2·3 | 9·4 | 52·3 | 0·1 | 4·4 | 5·3 | 6·0 | 64·8 |
| Age N.K. | 15,000 | 0 | 1 | 1 | 1 | 8 | 0 | 0 | 0 | 0 | 0 | 0 | 0 | 0 | 0 | 0 |
| All ages | 240,000 | 9 | 14 | 59 | 55 | 330 | 5·7 | 24·2 | 97·7 | 180·9 | 793·7 | 7·1 | 37·3 | 211·9 | 137·5 | 744·6 |

TABLE VII.—*Numbers of New Cases of Malignant Disease Diagnosed in Ibadan Residents (Both Sexes) April 1, 1960–March 31, 1963*

*Age and Sex Groups*

| International List No. 7th Revision | Site | N.K | | 0–4 | | 5–9 | | 10–14 | | 15–19 | | 20–24 | | 25–29 | | 30–34 | | 35–39 | | 40–44 | | 45–49 | | 50–54 | | 55–59 | | 60–64 | | 65–69 | | 70–74 | | 75+ | | Totals | |
|---|---|---|---|---|---|---|---|---|---|---|---|---|---|---|---|---|---|---|---|---|---|---|---|---|---|---|---|---|---|---|---|---|---|---|---|---|---|
| | | M. | F. | M. | F. | M. | F. | M. | F. | M. | F. | M. | F. | M. | F. | M. | F. | M. | F. | M. | F. | M. | F. | M. | F. | M. | F. | M. | F. | M. | F. | M. | F. | M. | F. | M. | F |
| 140 | Lip | — | — | — | — | — | — | — | — | — | — | — | — | — | — | — | — | — | — | — | — | — | — | — | — | — | — | 1 | — | — | — | — | — | — | — | 1 | — |
| 141 | Tongue | — | — | — | — | — | — | — | — | — | — | — | — | — | — | — | — | — | — | — | — | — | — | — | — | 1 | — | — | — | — | — | — | — | — | — | 1 | — |
| 142 | Salivary glands | — | — | — | — | — | — | — | — | — | — | — | — | — | — | — | — | — | — | 1 | 3 | 2 | — | — | — | — | — | 2 | — | — | — | — | — | — | — | 5 | 3 |
| 143–4 | Other Mouth | — | — | — | — | — | — | — | — | — | — | — | — | — | — | 1 | — | 1 | — | 1 | — | — | — | — | — | — | — | — | — | 1 | — | — | — | — | — | 4 | — |
| 146 | Nasopharynx | — | — | — | — | — | — | — | — | — | — | — | — | — | — | — | — | — | — | 1 | — | — | — | — | — | — | — | — | — | 1 | — | — | — | — | — | 2 | — |
| 145, 147, 148 | Other Pharynx | — | — | — | — | — | — | — | — | — | — | — | — | — | — | — | — | — | — | — | — | — | — | — | — | — | — | — | — | — | — | — | — | — | — | — | — |
| 150 | Oesophagus | 1 | — | — | — | — | — | — | — | — | — | — | — | — | — | — | — | — | — | — | 1 | — | — | 1 | — | — | — | 1 | — | — | — | — | — | — | — | 3 | 1 |
| 151 | Stomach | — | 1 | — | — | — | — | — | — | — | — | 1 | — | 1 | — | — | 2 | 1 | 1 | 4 | 2 | 6 | 3 | 3 | 1 | 3 | 1 | 4 | 2 | 4 | 1 | — | — | 1 | — | 28 | 14 |
| 152 | Duodenum, Small Intestine | — | — | — | — | — | — | — | — | — | — | — | — | — | — | — | — | — | — | — | — | — | — | — | — | — | — | — | — | — | — | — | — | — | — | — | — |
| 153 | Colon | — | — | — | — | — | — | — | — | — | — | — | — | — | — | 1 | — | — | — | 1 | 1 | 1 | 1 | — | 1 | 1 | — | — | — | — | — | — | 1 | — | — | 4 | 4 |
| 154 | Rectum | — | — | — | — | — | — | 1 | — | — | — | — | — | — | — | 2 | — | — | 1 | — | 1 | 2 | 2 | — | — | 1 | — | — | — | — | — | — | — | — | — | 6 | 4 |
| 155 | Liver, Primary | 1 | — | — | — | — | — | — | — | 2 | — | 3 | — | 4 | — | 4 | — | 7 | 2 | 8 | — | 3 | 3 | 2 | — | 3 | 3 | 5 | — | — | — | — | — | — | — | 42 | 8 |
| 155.1 | G.B. Extra Hepatic ducts | — | — | — | — | — | — | — | — | — | — | — | — | — | — | — | — | — | — | — | — | — | — | — | — | — | 1 | — | — | — | — | — | — | — | — | — | 1 |
| 156 | Liver 2° | — | 1 | — | — | — | — | — | — | — | — | — | — | — | — | — | — | 1 | — | — | — | — | — | — | 3 | — | — | — | — | 1 | — | — | — | — | — | 2 | 4 |
| 157 | Pancreas | — | — | — | — | — | — | — | — | — | — | 1 | — | 1 | — | 1 | — | 2 | 1 | 1 | — | 1 | 2 | 1 | 1 | — | 1 | — | — | — | — | — | — | — | — | 8 | 5 |
| 160 | Nasal sinuses | — | — | 1 | — | — | — | — | — | — | — | 1 | — | — | — | 1 | — | 1 | — | 1 | — | 2 | — | 1 | — | — | — | — | — | — | — | — | — | — | — | 8 | — |
| 161 | Larynx | — | — | — | — | — | — | — | — | — | — | — | — | — | — | — | — | — | — | 1 | — | — | — | 1 | — | 1 | — | 1 | — | — | — | — | — | — | — | 4 | — |
| 162, 163 | Bronchus, Lung | — | — | — | — | — | — | — | — | — | — | 1 | — | — | — | 1 | — | 1 | 2 | — | — | — | 2 | 1 | — | — | — | 1 | 1 | — | — | — | — | — | — | 5 | 5 |
| 170 | Breast | — | 1 | — | — | — | — | — | — | — | — | — | — | — | 4 | — | 4 | — | 11 | — | 7 | — | 4 | — | 14 | — | 4 | — | 3 | — | 3 | — | — | — | — | — | 55 |
| 171 | Cervix uteri | — | 1 | — | — | — | — | — | — | — | — | — | — | — | — | — | — | — | 8 | — | 8 | — | 13 | — | 13 | — | 8 | — | 3 | — | 3 | — | 1 | — | 1 | — | 59 |
| 173 | Other specific U.T. including Chorionepithelioma | — | — | — | — | — | — | — | — | — | 1 | — | 3 | — | 2 | — | 2 | — | 4 | — | 3 | — | — | — | — | — | — | — | — | — | — | — | — | — | — | — | 15 |
| 174 | Unspecific Uterus | — | — | — | — | — | — | — | — | — | — | — | — | — | — | — | — | — | — | — | — | — | — | — | — | — | — | — | — | — | — | — | — | — | — | — | — |
| 175 | Ovary | — | — | — | — | — | 1 | — | — | — | — | — | 1 | — | 5 | — | 4 | — | 2 | — | 4 | — | 1 | — | 6 | — | 4 | — | 2 | — | 1 | — | — | — | — | — | 31 |
| 176 | Other F. Genital | — | — | — | — | — | — | — | — | — | — | — | — | — | — | — | — | — | — | — | — | — | — | — | 2 | — | — | — | — | — | — | — | — | — | — | — | 2 |
| 177 | Prostate | — | — | — | — | — | — | — | — | — | — | — | — | — | — | — | — | — | — | — | — | 1 | — | 2 | — | 4 | — | 4 | — | 4 | — | 3 | — | — | — | 18 | — |
| 178 | Testis | 1 | — | — | — | — | — | — | — | — | — | 2 | — | — | — | — | — | — | — | — | — | — | — | — | — | — | — | — | — | — | — | — | — | — | — | 3 | — |
| 179 | Penis | — | — | — | — | — | — | — | — | — | — | — | — | — | — | — | — | — | — | — | — | — | — | 1 | — | — | — | — | — | — | — | — | — | — | — | 1 | — |
| 180 | Kidney | — | — | 2 | 1 | 1 | — | — | — | — | — | — | — | — | — | — | — | — | 1 | — | — | — | — | 1 | — | — | — | — | — | — | — | — | — | — | — | 4 | 2 |
| 181.0 | Bladder | — | — | — | — | — | — | — | — | — | 1 | — | — | — | — | — | — | — | — | 3 | — | — | 1 | 1 | 2 | — | 1 | 4 | — | 1 | — | 1 | — | — | — | 10 | 5 |
| 190 | Melanoma of skin | — | — | — | — | — | — | — | — | — | — | 1 | — | — | — | — | — | 1 | — | — | — | — | 1 | — | 1 | — | — | — | — | 1 | 1 | — | — | — | — | 3 | 3 |
| 191 | Other skin | 2 | — | — | — | — | — | — | — | — | — | 1 | 1 | 1 | — | 1 | — | — | — | — | — | 1 | 1 | — | — | — | — | 1 | 1 | — | — | — | — | — | — | 7 | 3 |
| 192 | Eye | — | — | 3 | 1 | 1 | — | — | — | — | — | — | — | — | — | — | — | — | — | — | — | — | — | — | — | — | — | — | — | — | — | — | — | — | — | 4 | 1 |
| 193, 223, 227 | Brain C.N.S. | — | — | 1 | 2 | 2 | — | — | — | 1 | — | — | — | — | — | — | — | — | — | — | — | — | — | — | — | — | — | — | — | 1 | — | — | — | — | — | 5 | 2 |
| 194 | Thyroid | — | 2 | — | — | — | — | — | 1 | — | — | 2 | 1 | — | 1 | — | — | 1 | 1 | 2 | 1 | — | — | — | 3 | — | — | — | — | — | 1 | — | — | — | — | 5 | 11 |
| 195 | Other Endocrines | — | — | — | — | — | — | — | — | — | — | — | — | — | — | — | — | — | — | — | — | — | — | — | — | — | — | — | — | — | — | — | — | — | — | — | — |
| 196 | Bone | — | — | — | — | — | 1 | — | — | — | 1 | 1 | 1 | 4 | — | — | — | — | — | 1 | — | 1 | — | — | — | — | — | — | — | — | — | — | — | — | — | 7 | 3 |
| 197 | Connective tissue + muscle | 1 | — | 2 | — | — | — | — | — | 2 | 1 | — | 1 | 2 | 1 | — | — | — | 1 | — | — | 1 | — | — | 1 | — | — | — | — | — | 1 | — | — | — | — | 8 | 6 |
| 200 | Lymphosarcoma + Reticulosarcoma | — | 1 | 1 | — | — | — | — | — | 1 | — | 3 | 3 | — | 5 | 4 | 1 | 3 | 2 | 3 | 4 | 1 | 2 | 4 | 5 | 2 | 2 | — | 1 | 1 | — | — | — | — | — | 23 | 26 |
| 200.1 | Burkitt's tumour | — | — | 3 | 1 | 19 | 10 | 11 | 7 | 1 | 2 | 1 | — | 1 | — | — | — | — | — | — | — | — | — | — | — | — | — | — | — | — | — | — | — | — | — | 36 | 20 |
| 201 | Hodgkin's disease | — | — | — | — | — | — | 2 | — | 2 | — | 2 | — | 1 | — | 1 | — | — | — | 2 | — | 1 | 1 | 1 | — | — | 1 | — | — | 1 | — | — | — | — | — | 13 | 2 |
| 203 | Multiple Myeloma | — | — | — | — | — | — | — | — | — | — | — | — | — | — | — | — | 1 | — | — | — | — | — | — | — | — | — | — | — | — | — | — | — | — | — | 1 | — |
| 204 | Leukaemia | — | — | 1 | — | 1 | 2 | 5 | — | 2 | — | — | — | — | 4 | 3 | 1 | 5 | — | — | 1 | 4 | 2 | — | 3 | 1 | — | 1 | 3 | 1 | — | — | — | — | — | 24 | 16 |
| 198 | 2° Tumours in L. Glands | 2 | — | — | — | — | — | — | — | — | — | — | — | — | 1 | — | — | — | 1 | 2 | 2 | — | — | 1 | — | — | — | — | — | 1 | — | — | — | — | — | 6 | 4 |
| 199 | 2° Tumours, unspecific 1° 1° Site | — | 1 | — | — | 1 | — | — | — | — | — | — | — | 1 | — | 2 | 2 | — | 1 | 3 | 3 | 2 | 1 | 3 | 3 | — | 1 | 3 | 2 | 2 | 1 | — | — | — | — | 17 | 15 |
| | Totals | 8 | 8 | 14 | 5 | 25 | 14 | 19 | 8 | 11 | 6 | 20 | 11 | 16 | 23 | 22 | 16 | 25 | 39 | 35 | 41 | 29 | 40 | 24 | 59 | 17 | 27 | 28 | 18 | 20 | 12 | 4 | 2 | 1 | 1 | 318 | 330 |

tumour is high and accounts for the relatively high incidence of malignant disease in the 5–14 year old age group (Edington and Maclean, 1964) and the incidence of primary liver celled carcinoma is also much higher than would be expected in the United States. Detailed analyses of other individual tumour types are at present being undertaken but, whilst it is acknowledged that a low incidence of a certain type of malignancy may in itself suggest valuable aetiological information, there is no doubt that the two greatest problems in cancer research in Ibadan are the Burkitt tumour and primary liver celled carcinoma.

SUMMARY

The methods employed in a cancer rate survey in Ibadan, Western Nigeria, have been described. The incidence of malignant disease in Ibadan is similar to that recorded in the United States until the age of 50 years in males and 20 years in females. Over these ages malignant disease is much less common in the Nigerian. Particular attention is drawn to the actual fall in cancer incidence in the elderly in Ibadan which appears to occur a decade or two earlier than it does in the United States non-white population. The incidence of four of the most common tumours occurring in Ibadan (carcinoma of breast, cervix, liver and stomach) has been compared with the recorded incidence in the United States white and non-white populations.

We are most grateful to the British Empire Cancer Campaign for Research who supported the salary of one of us (C. M. U. Maclean) and made this investigation possible. Grateful acknowledgement must also be made to the medical and nursing staff in the University College and Adeoyo Hospitals for their forbearance and cooperation over the years. To the Medical Records Department and Medical Illustration Unit we are also specially indebted. Professor J. Knowelden gave most valuable advice at the commencement of this investigation and to him also we are grateful.

### REFERENCES

DAVIES, J. N. P., WILSON, B. A. AND KNOWELDEN, J.—(1962) *Lancet*, ii, 328.

DORN, H. F. AND CUTLER, S. J.—(1955) 'Morbidity from Cancer in the United States.' Public Health Monograph No. 29. Washington (U.S. Government Printing Office).

EDINGTON, G. M.—(1964) International Academy of Pathology Meeting. June. London (in press).

EDINGTON, G. M. AND MACLEAN, C. M. U.—(1964) *Brit. med. J.*, i, 264.

HIGGINSON, J. AND OETTLÉ, A. G.—(1957) *Acta Un. int. Cancr.*, **13**, 949.

'International Statistical Classification of Diseases, Injuries and Causes of Death.' (1948) Vol. 1, World Health Organisation, Geneva.

KNOWELDEN, J. AND OETTLÉ, A. G.—(1962) Unpublished data used by Davies, J. N. P., Wilson, B. A. and Knowelden, J. (1962) *Lancet*, ii, 328.